# *Actinidia chinensis* Planch. Improves the Indices of Antioxidant and Anti-Inflammation Status of Type 2 Diabetes Mellitus by Activating Keap1 and Nrf2 via the Upregulation of MicroRNA-424

**DOI:** 10.1155/2017/7038789

**Published:** 2017-05-31

**Authors:** Longfeng Sun, Xiaofei Li, Gang Li, Bing Dai, Wei Tan

**Affiliations:** ^1^Department of Geriatrics, The First Affiliated Hospital of China Medical University, Shenyang 110001, China; ^2^Department of Emergency Medicine, The First Affiliated Hospital of China Medical University, Shenyang 110001, China; ^3^Department of Urology, Liaoning Cancer Hospital and Institute, Shenyang 110042, China

## Abstract

The fruit juice of *Actinidia chinensis* Planch. has antioxidant and anti-inflammation properties on patients with type 2 diabetes mellitus (T2DM), but the molecular mechanism was unclear. The patients took the juice and the serum level of antioxidant miR-424, Kelch-like ECH-associated protein 1 (Keap1), erythroid-derived 2-like 2 (Nrf2), and biochemical indices were measured. The juice increased the levels of serum microRNA-424, Keap1, and Nrf2 and reduced the levels of interleukin-1 (IL-1) beta and IL-6 in T2DM patients. The levels of SOD and GSH were higher while the levels of ALT and AST were lower in the patients consuming the juice when compared to the patients without taking the juice. The Spearman rank correlation analysis showed that the serum levels of miR-424 were positively related to Keap1 and Nrf2 levels while Keap1 and Nrf2 levels were positively related to the levels of SOD and GSH and negatively related to IL-1 beta and IL-6. Thus, FJACP improves the indices of antioxidant and anti-inflammation status by activating Keap1 and Nrf2 via the upregulation of miR-424 in the patients with T2DM. This trial is registered with ChiCTR-ONC-17011087 on 04/07/2017.

## 1. Introduction

Type 2 diabetes mellitus (T2DM) is increasingly prevalent in the elderly and has become a major public health issue. The nutrients of different foods will affect physiological activities and their imbalance may contribute to the development of T2DM. Thus, different diets will affect the risk of T2DM. The food is rich with fruits, vegetables, grains, and fish and low-level meat and fat may be beneficial to reduce the progression of T2DM [1]. Most diabetic patients cannot meet the demanded food and nutrients for a healthy person. For instance, some fruits are very important to health, but they cannot be used by most T2DM patients because most fruits have high-level sugar. The uptake of some vegetables and fruits has been found to be related to a low incidence of diabetes. However, fruit juice consumption can be harmful to increase the risk of diabetes in women [2].

Kiwi fruit (*Actinidia chinensis* Planch.) as a food with significant effects on human health, including antioxidant and anti-inflammatory activity, can inhibit the development and deterioration of the disorders caused by oxidative stress [3]. Lacking vitamin C is an important risk for the development of diabetes [4]. The vitamin C in *Actinidia chinensis* Planch. can reach up to 380 mg/100 g [5]. The daily uptake of vitamin C at a high level has been found to increase antisenescence and antiatherosclerotic activity by improving biochemical indices via antioxidant and antiglycation activities [6]. The risk of type 2 diabetes mellitus (T2DM) is often associated with muscle atrophy while inflammatory status will aggravate the symptoms. Clinical trial shows that vitamin C consumption has preventative and therapeutic functions for inflammatory symptoms in patients [7]. FJACP (fruit juice of *Actinidia chinensis* Planch.) has been found to improve physiological functions in the patients with neuropathy disorders [8]. More importantly, the main characters of FJACP are with low-level sugar content [9]. Thus, it may be useful for preventing the progression of T2DM. However, the related molecular mechanisms for the functions of FJACP on T2DM patients remain widely unknown.

Obesity patients with chronic low-grade inflammation have more chances to get heart disorders and T2DM. The etiology of this obesity-related proinflammatory is more focused on adipose tissue dysfunctions and/or insulin resistance in skeletal muscles [10]. Furthermore, inflammation has also been found to be associated with the development of insulin resistance in T2DM patients [11]. On the other hand, oxidative stress is also an important factor in the pathogenesis of T2DM complications. Alloxan-induced diabetes can cause an imbalance in an antioxidative system in the skeletal muscles of an animal model. Controlling the damage from oxidation stresses is also a potential therapeutic approach to prevent T2DM development [12]. These results suggest that inflammatory and oxidant stresses are the two main reasons for causing the impairment in T2DM patients. MicroRNAs (miRNAs), small, noncoding RNA, have been found to regulate protein expression and multiple cellular physiological activities, including antioxidant activities [13, 14]. Studies have shown that miR-424 plays a critical role in the antioxidant activity by suppressing oxidant stress. MiR-424 therapy prevents infarct volume and neuronal apoptosis, reduces the levels of reactive oxygen species (ROS) and malondialdehyde (MAD), and enhances the activities of the antioxidant enzyme SOD. All the results suggest that miR-424 has protective effects against oxidative stress [15]. On the other hand, the nuclear factor, erythroid-derived 2-like 2 (Nrf2), is a redox-sensitive transcription factor and related with inflammatory disease [16, 17]. Nrf2 can combine with Kelch-like ECH-associated protein 1 (Keap1) to form an important complex to increase the levels of antioxidant molecules [18]. However, the relationship between the serum level of miR-424 and antioxidants Keap1 and Nrf2 remains unclear. Therefore, we want to explore the effects of FJACP on Keap1 and Nrf2 in T2DM patients by investigating the serum levels of miR-424.

## 2. Materials and Methods

### 2.1. Participants

All protocols were approved by the Ethical Committee of The First Affiliated Hospital of China Medical University (Shenyang, China), and the study was carried out according to the principles described in the World Medical Association Declaration of Helsinki [19]. A total of 122 patients, at the age of 50–70, were recruited from our hospital and diagnosed with T2DM.

### 2.2. Including Criteria

The following including criteria were used: (1) the patients seldom consumed fruits daily; (2) the patients had normal work and spouse; (3) they wanted to join our research, such as receiving FJACP treatment; (4) the patients were diagnosed with T2DM; (5) the patients had a body mass index (BMI) more than 25 and less than 39 kg/m^2^; and (6) their weight were stable within three months before the present experiment.

### 2.3. Excluding Criteria

The following excluding criteria were performed: (1) the patients had senile dementia, such as Parkinson's disease (PD), Alzheimer's disease (AD), and brain injury; (2) the patients suffered from cardiac disorder, hypertension, dizziness, and related disorders; (3) the patients had the obstacle for limb movement; and (4) the patients had obvious abnormal clinical findings.

### 2.4. Study Design

**Figure 1:**
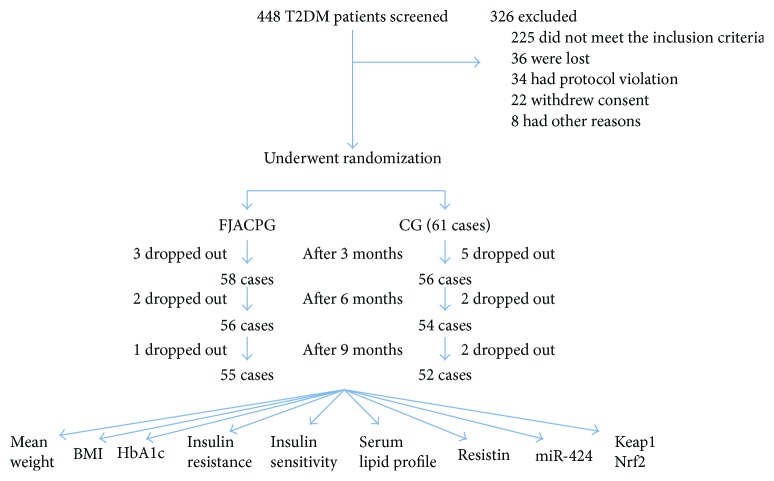
The flowchart of the present study. FJACPG: FJACP group; CG: placebo group. The whole period of the follow-up was nine months.

After the selection by using the including criteria and excluding criteria, 122 patients were evenly and randomly assigned into a FJACP group (FJACPG, received 10 ml FJACP daily) and a control group (CG, received 10 ml liquid placebo daily) (Figure 1). The whole period was nine months. Exercise (each person walks about 1.5 to 2 miles per day) was considered for both groups at the same level. Fifteen patients (6 patients from FJACPG and 9 patients from CG) dropped out at the end of the experiment. Among the patients, six patients dropped out of FJACPG and nine patients dropped out of CG. Thus, 55 and 52 patients from FJACPG and CG finished the whole experiment.

### 2.5. Biochemical Analysis

All the patients had a random capillary glucose level more than 6.1 mM and regarded as higher risk of diabetes [20] and needed further examination. The concentrations of glucose and HbAlc were tested after a 2-hour 75-gram oral glucose intake. Glucose concentrations were measured by using YSI 2700 Select Biochemical Analyzer (YSI, Yellow Springs, OH, USA). HbA1c levels were measured by BIO-RAD D-10 HPLC (Hercules, CA, USA). High- and low-density lipoprotein-cholesterol was measured by using HDL and LDL/VLDL Cholesterol Assay Kit (Cat. number ab65390, Abcam Shanghai office launch, Shanghai, China). Triglyceride levels were measured by using a Triglyceride Quantification Assay Kit (ab65336). Basal blood glucose (BG) and fasting blood glucose (FBG) levels were measured by ABL800 FLEX blood gas analyzer (Midland, ON, Canada). Serum basal insulin (BINS) and fasting insulin (FINS) were tested by radioimmunoassay (Linco, Seaford, DE, USA). Insulin resistance (HOMA − IR = FBG × FINS/22.5) and insulin secretory function (HOMA − IS = 20 × FINS/(FBG − 3.5) were calculated.

### 2.6. Analysis of Enzyme Activities

Diabetes is associated with increased oxidative stress [21, 22]. SOD [23, 24], AST [25, 26], ALT [27, 28], and GSH are associated with oxidative stress [29–34]. Here, the levels of SOD, AST, ALT, and GSH were measured. AST and ALT were measured by an automated clinical chemistry analyzer (Indianapolis, IN, USA). The activity of SOD was measured by Superoxide Dismutase Activity Assay Kit (Cat. number ab65354 from Abcam). GSH was measured by Glutathione Detection Assay Kit (Cat. number ab65322 from Abcam).

### 2.7. Measurement of Lipid Pattern Indexes

The serum lipid pattern, including total triglycerides (TG), total cholesterol (TC), high-density lipoprotein-cholesterol (HDL-C), and low-density lipoprotein-cholesterol (LDL-C), was measured by FACA-401 Fully Automatic Biochemistry Analyzer (Labomed Inc., Los Angeles, CA, USA). Malondialdehyde (MDA) level was measured by using a Lipid Peroxidation (MDA) Assay Kit (Sigma, St. Louis, MO, USA). Lipid indexes were measured before and after 3 months of the present experiment.

### 2.8. Measurement of Inflammatory Cytokines

The pathology of diabetes is closely associated with interleukin-1 (IL-1) beta and IL-6. 5 ml blood samples were obtained from all participants before the experiment, and after 3-, 6-, and 9-month exercises. Thus, the levels of these cytokines were measured by using human ELISA kits for IL-1 beta (Cat. number ab100562) and IL-6 (Cat. number ab46042) and Nrf2 Transcription Factor Assay Kit (ab207223) from Abcam (Shanghai) Ltd. (Shanghai, China).

Serum inflammatory cytokines were measured before and after 3, 6, and 9 months of the present experiment.

### 2.9. Quantitative Real-Time RT-PCR (qRT-PCR)

RNA was isolated from blood samples by a RNA purification kit (Thermo Fisher Scientific, Waltham, MA, USA). cDNA was synthesized by cDNA Synthesis Kits from Thermo Fisher Scientific. SYBR Green Real-Time PCR Master Mixes (Thermo Fisher Scientific) was used for qRT-PCR. The following primers were synthesized: miR-424, F: 5′-cgaggggatacagcagcaat-3′, R: 5′- ccccaccttctaccttcc (98 bp); Keap1, F: 5′-gaggtgacgccctcccagca-3′, R: 5′-catggccttgaagacagggc-3′ (210 bp); Nrf2, F: 5′- agtggatctgccaactactc-3′, R: 5′-agtgactgaaacgtagccga-A3′ (140 bp); and GAPDH, F: 5′-GGAAAGCFJACPGFJACPGGCGFJACPGAT-3′, R: 5′-AAGGFJACPGGAAGAAFJACPGGGAGTT-3′. The values of cycle time for the interest genes were normalized with GAPDH.

### 2.10. Western Blot Analysis

Total proteins were extracted by using a total protein isolation kit (ITSI-Biosciences, Johnstown, PA, USA). Total proteins were separated by SDS-PAGE and transferred to a polyvinylidene difluoride (PVDF) (RTP Company, Winona, MN, USA). The PVDF was incubated with rabbit polyclonal Keap1 antibody (ab139729, Abcam), rabbit polyclonal anti-Nrf2 antibody (ab137550, Abcam), and rabbit polyclonal GAPDH antibody (ab37168, Abcam) overnight at 4°C. Subsequently, the sample was incubated with peroxidase and peroxidase-conjugated goat anti-rabbit IgG (ab97051, Abcam). The immunoreactive bands were visualized with DAB (Sigma-Aldrich Chemicals, St. Louis, MO, USA) and densitometry was quantified by ImageJ software (National Institutes of Health, Bethesda, MD, USA).

### 2.11. Statistical Analysis

SPSS20.0 was used to process the data. Adopt t to detect when comparing the measurement data. Spearman's rank correlation was used for the comparison of two variables. Results were presented as the mean ± SD. In the case of *P* < 0.05, there were significantly statistical differences.

## 3. Results

### 3.1. Baseline Characters

Table 1 showed the clinical characters were similar between the two groups. All the patients were at the age of 56.1 ± 14.4 in FJACPG and 57.5 ± 12.3 in CG. The ratio of males and females was 37/24 and 39/22 in FJACPG and CG, respectively. There was no significantly statistical difference for baseline demographic and metabolic characteristics of the patients between the two groups (*P* > 0.05).

### 3.2. The Changes for Biochemical Indexes between the Two Groups

Table 2 showed FJACPG and CG could not improve insulin resistance and insulin secretory function. FJACP increased the level of HDL and reduced the levels of TG, TC, and LDL-C (*P* < 0.05). There were significant statistical differences in these parameters between the two groups (*P* < 0.05).

### 3.3. Biochemical Parameters of Enzyme Activities

The levels of SOD, AST, ALT, and GSH were measured. As showed in Table 3, FJACP and control groups increased SOD and GSH levels after 3 months when compared with the levels before this study (*P* < 0.05). There was no significant statistical difference for these parameters between the two groups after 3-month therapy (*P* > 0.05). The levels of SOD and GSH were higher in FJACPG than in CG after 6-month therapy (*P* < 0.05). Similarly, the levels of SOD and GSH were higher in FJACPG than in CG after 9-month therapy (*P* < 0.05). In contrast, the levels of ALT were lower in FJACPG than CG after 9 month therapy (*P* < 0.05). The results suggested that long-term FJACP consumption showed better results for antioxidant activities when compared with CG.

### 3.4. Comparison of Lipid Pattern

Table 4 showed that the serum levels of TG, TC, LDL-C, and MDA were reduced while the level of HDL-C was increased in FJACPG after 3-month therapy, but there was no statistical significance of difference between the two groups (*P* > 0.05). Lipid pattern was improved further in FJACPG after 6-month therapy, and there was statistical significance of differences between the two groups (*P* < 0.05). Similarly, lipid pattern was improved significantly in FJACPG after 9 months, and there was statistical significance of difference between the two groups (*P* < 0.05). All the results suggested that FJACP significantly improved lipid patterns in T2DM patients.

### 3.5. Long-Term FJACP Significantly Reduces the Levels of IL-1 Beta and IL-6

**Figure 2:**
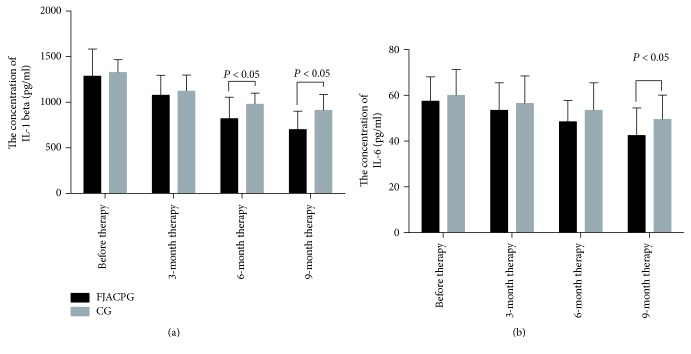
Measurement of the concentrations of IL-1 beta and IL-6 by ELISA in blood samples. (a) The concentration of IL-1 beta. (b) The concentration of IL-6. FJACPG: FJACP group; CG: placebo group. All data were presented as mean values ± SD.

As Figure 2 showed, before this study, there was no significant statistical difference in blood concentrations of IL-1 beta and IL-6 between the two groups (*P* > 0.05). Comparatively, the levels of IL-1 beta and IL-6 were reduced in both groups (*P* < 0.05), but there was still no significantly statistical difference in blood concentrations of IL-1 beta and IL-6 between the two groups after 3-month therapy (*P* > 0.05). The levels of IL-1 beta and IL-6 were further reduced in both groups (*P* < 0.05), and there were significant statistical differences in blood concentrations of IL-1 beta between the two groups after 6- and 9-month therapy (Figure 2(a), *P* < 0.05). There were significant statistical differences for blood concentrations of IL-6 between the two groups after and 9-month therapy (Figure 2(b), *P* < 0.05). The results suggest that long-term FJACP significantly reduces the levels of IL-1 beta and IL-6.

### 3.6. Long-Term FJACP Consumption Increased the Levels of Serum miR-424, Keap1, and Nrf2

**Figure 3:**
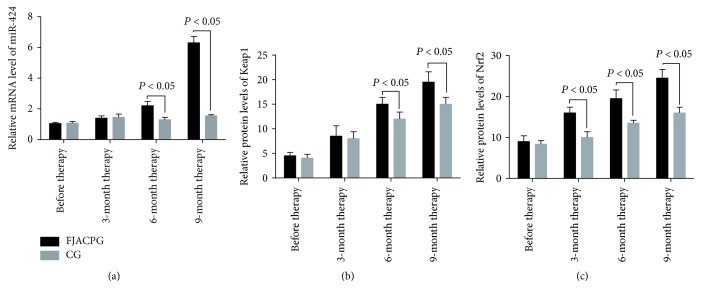
qRT-PCR analysis of relative mRNA levels. (a) Relative mRNA levels of serum miR-424. (b) Relative mRNA levels of Keap1. (c) Relative mRNA levels of Nrf2. FJACPG: FJACP group; CG: placebo group. All data were presented as mean values ± SD.

As Figure 3 showed, before this study, there was no significant statistical difference for mRNA levels of serum miR-424, Keap1, and Nrf2 between the two groups (*P* > 0.05). Comparatively, mRNA levels of serum miR-424, Keap1, and Nrf2 were increased in both groups (*P* < 0.05), but there were significantly statistical differences for mRNA levels of Nrf2 between the two groups after 3 months (*P* < 0.05). The mRNA levels of serum miR-424, Keap1, and Nrf2 were further increased in both groups (*P* < 0.05), and there were significant statistical differences in mRNA levels of Keap1 and Nrf2 between the two groups since 6 months (Figure 3, *P* < 0.05). Much difference for mRNA levels of serum miR-424, Keap1, and Nrf2 was observed between the two groups after and 9 months (Figures 3(a), 3(b), and 3(c), *P* < 0.05). The results suggest that long-term FJACP consumption significantly increases mRNA levels of Keap1 and Nrf2.

### 3.7. Long-Term FJACP Consumption Increased Protein Levels of Keap1 and Nrf2

**Figure 4:**
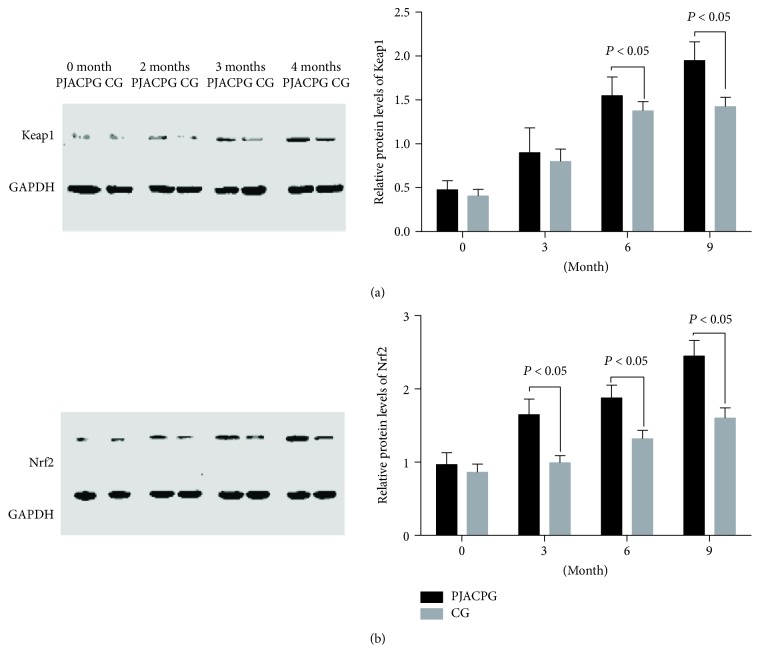
Western blot analysis of relative protein levels of Keap1 and Nrf2. (a) Relative protein levels of Keap1. (b) Relative protein levels of Nrf2. FJACPG: FJACP group; CG: placebo group. All data were presented as mean values ± SD.

As Figure 4 showed, before this study, there was no significantly statistical difference for protein levels of Keap1 and Nrf2 between the two groups (*P* > 0.05). Comparatively, the protein levels of Keap1 and Nrf2 were increased in both groups (*P* < 0.05), but there were only significant statistical differences for the protein levels of Nrf2 between the two groups after 3-month therapy (*P* < 0.05). The protein levels of Keap1 and Nrf2 were further increased in both groups (*P* < 0.05), and there were significant statistical differences in the protein levels of Keap1 and Nrf2 between the two groups since 6-month therapy (Figure 4, *P* < 0.05). Much difference for the protein levels of Keap1 and Nrf2 was observed between the two groups after and 9-month therapy (Figures 4(a) and 4(b), *P* < 0.05). The results suggest that long-term FJACP significantly increases protein levels of Keap1 and Nrf2.

### 3.8. The Association of the Levels between miR-424 and Inflammatory Cytokines

**Figure 5:**
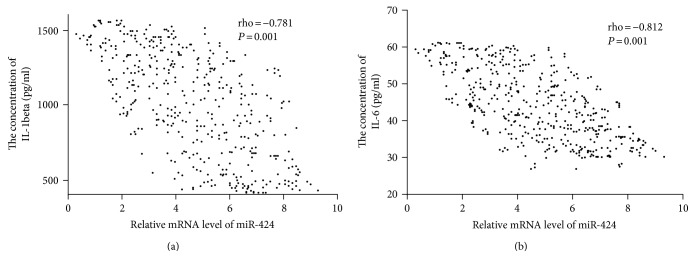
The relationship between the level of miR-424 and the levels of proinflammatory cytokines. (a) The relationship between the level of miR-424 and the level of IL-1 beta. (b) The relationship between the level of miR-424 and the level of IL-6. Spearman's rank correlation test was performed to compare two variables. There is a strong negative association if the value of rho falls between −1 and −0.5.

Spearman's rank correlation analysis showed that the levels of IL-1 beta (Figure 5(a)) and IL-6 (Figure 5(b)) were reduced when the level of miR-424 was increased. There was a negative relationship between the level of miR-424 and the level of IL-1 beta or IL-6 (*P* < 0.05). The results suggest that a higher level of Nrf2 will result in lower levels of IL-1 beta and IL-6.

### 3.9. The Association of the Levels between miR-424 and Biomarkers of Antioxidant Factors

**Figure 6:**
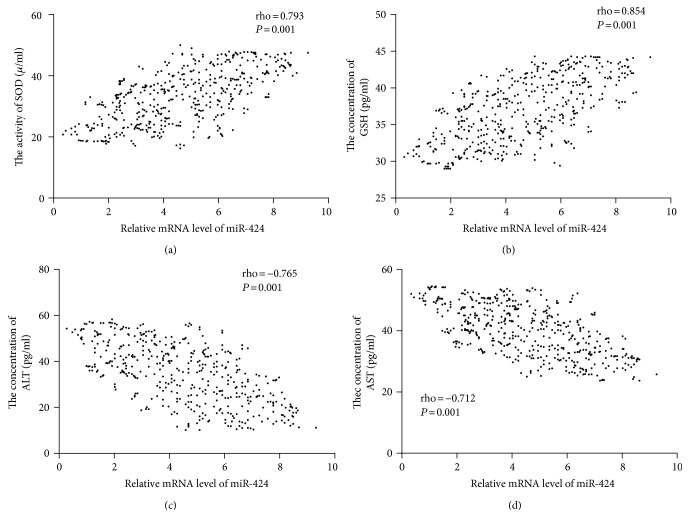
The relationship between the level of miR-424 and the levels of biomarkers of antioxidant factors. (a) The relationship between the level of miR-424 and the level of SOD. (b) The relationship between the level of miR-424 and the level of GSH. (c) The relationship between the level of miR-424 and the level of ALT. (d) The relationship between the level of miR-424 and the level of AST. Spearman's rank correlation test was performed to compare two variables. There is a strong negative association if the value of rho falls between −1 and −0.5. There is a strong positive association if the value of rho falls between 0.5 and 1.

Spearman's rank correlation analysis showed that the levels of SOD (Figure 6(a)) and GSH (Figure 6(b)) were increased when the level of miR-424 was increased. There was a positive relationship between the levels of miR-424 and the levels of IL-1 beta or IL-6 (*P* < 0.05). In contrast, the levels of ALT (Figure 6(c)) and AST (Figure 6(d)) were reduced when the level of miR-424 was increased. The results suggest that a higher level of miR-424 will promote antioxidant activities of T2DM patients.

### 3.10. The Association of the Protein Levels between Nrf2 and Inflammatory Cytokines

**Figure 7:**
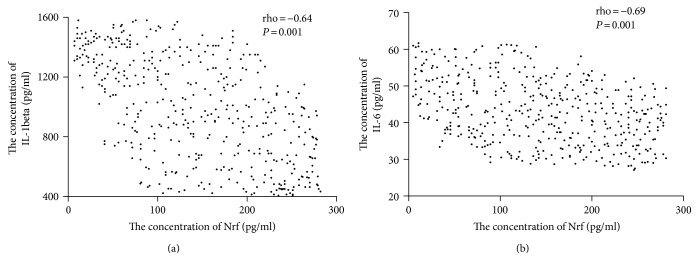
The relationship between the level of Nrf2 and the levels of proinflammatory cytokines. (a) The relationship between the level of Nrf2 and the level of IL-1 beta. (b) The relationship between the level of Nrf2 and the level of IL-6. Spearman's rank correlation test was performed to compare two variables. There is a strong negative association if the value of rho falls between −1 and −0.5.

Spearman's rank correlation analysis showed that the levels of IL-1 beta (Figure 7(a)) and IL-6 (Figure 7(b)) were reduced when the level of Nrf2 was increased. There were a negative relationship between the level of Nrf2 and the level of IL-1 beta or IL-6 (*P* < 0.05). The results suggest that a higher level of Nrf2 will result in lower levels of IL-1 beta and IL-6.

### 3.11. The Association of the Protein Levels between Nrf-2 and Biomarkers of Antioxidant Factors

**Figure 8:**
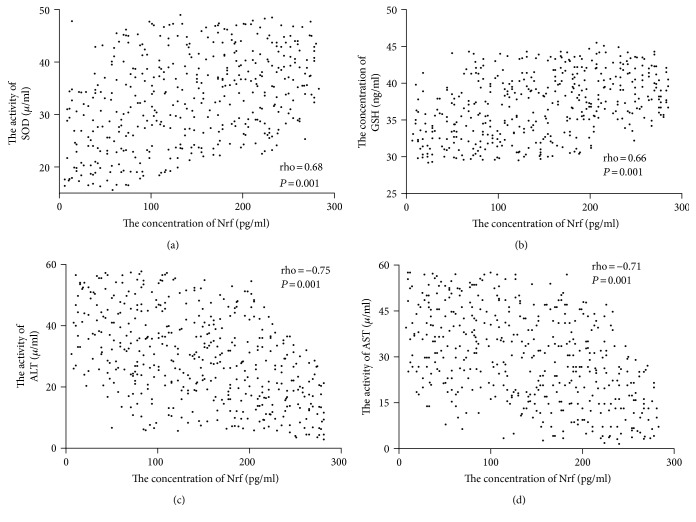
The relationship between the level of Nrf2 and the levels of biomarkers of antioxidant factors. (a) The relationship between the level of Nrf2 and the level of SOD. (b) The relationship between the level of Nrf2 and the level of GSH. (c) The relationship between the level of Nrf2 and the level of ALT. (d) The relationship between the level of Nrf2 and the level of AST. Spearman's rank correlation test was performed to compare two variables. There is a strong negative association if the value of rho falls between −1 and −0.5. There is a strong positive association if the value of rho falls between 0.5 and 1.

Spearman's rank correlation analysis showed that the levels of SOD (Figure 8(a)) and GSH (Figure 8(b)) were increased when the level of Nrf2 was increased. There was positive relationship between the level of Nrf2 and the level of IL-1 beta or IL-6 (*P* < 0.05). In contrast, the levels of ALT (Figure 8(c)) and AST (Figure 8(d)) were reduced when the level of Nrf2 was increased. The results suggest that a higher level of Nrf2 will promote antioxidant activities of T2DM patients.

### 3.12. The Association of the Levels between miR-424 and Nrf2

**Figure 9:**
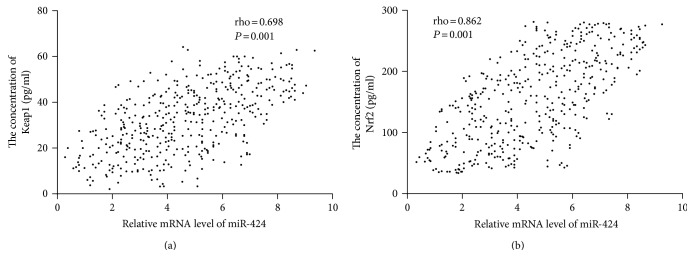
The relationship between the level of miR-424 and the levels of Keap1 or Nrf2. (a) The relationship between the level of miR-424 and the level of Keap1. (b) The relationship between the level of miR-424 and the level of Nrf2. Spearman's rank correlation test was performed to compare two variables. There is a strong negative association if the value of rho falls between −1 and −0.5. There is a strong positive association if the value of rho falls between 0.5 and 1.

Spearman's rank correlation analysis showed that the levels of Keap1 (Figure 9(a)) and Nrf2 (Figure 9(b)) were increased when the level of miR-424 was increased. There was a positive relationship between the level of miR-424 and the level of Keap1 or Nrf2 (*P* < 0.05). The results suggest that a higher level of miR-424 will increase the levels of Keap1 and Nrf2.

## 4. Discussion

The changes of physical indexes indicated that both the control group and the FJACP group improved the antioxidant activities of T2DM. Notably, long-term FJACP showed a better result after 3-month therapy. For the most elderly, the ability of movement begins to decline and the strength of the skeletal muscles of the lower limbs plays a critical role in the activity. In the population cohort with difficult walking, higher risk of diabetic complications will be developed [35]. In contrast, long-term consumption of FJACP significantly ameliorates diabetes, including the improvement of general well-being and the decrease of the levels of HbA1c, FBG, and BMI [36]. Similarly, FJACP also has been reported to have the functions for reducing the levels of BMI [37] and blood glucose [38].

Present comparing study showed physical condition was improved in both groups after 3 months. Furthermore, the knee extension strength and balance function in FJACPG were improved obviously (data was not shown). The results might be caused by the normal exercise (walking daily) which could be maintained. The exercise was kept at the same level (each person walks about 1.5 to 2 miles per day) between the two groups. The exercise function was not explored in detail. The general improvement in the patients from FJACPG was better than those from CG. After 6 months, there were significant statistical differences between FJACPG and CG. The results suggest that FJACP may have some benefits on general well-being. The work is not the focus of the present study, but it is very important and will be performed in the future to confirm the conclusion.

FJACP improves the indices of antioxidant and anti-inflammation status by affecting Keap1 and Nrf2 in the patients with T2DM. Keap1 and Nrf2 play an important role in preventing the risk and development of T2DM. It can reduce the levels of proinflammatory cytokines and increase the level of antioxidant molecules. T2DM is characterized by inflammatory [39, 40] and oxidant status [41, 42]. The disease is closely associated with kidney disorder [43, 44] and peripheral neuropathy [45, 46]. T2DM is also the main cause for cardiovascular disorder [47, 48]. The levels of Nrf2 and Keap1 were significantly decreased in T2DM patients. To make sure the interaction between Nrf2 and Keap1, and these molecules, is present, much work is needed to be done in future work.

**Figure 10:**
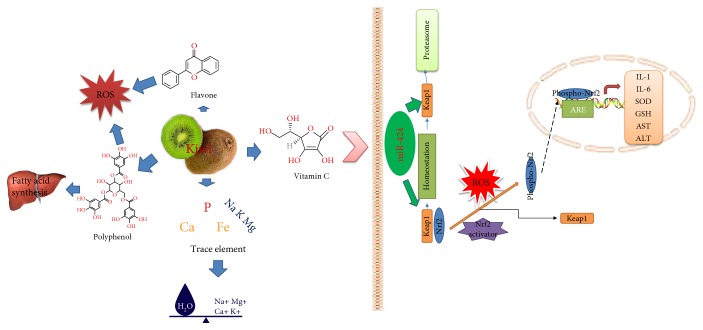
The multiple functions of FJACP on T2DM patients.

The reasons for the functions of FJACP are complex (Figure 10). The reasons for the functions of FJACP are complex (Figure 10). FJACP contains a large number of antioxidants such as polyphenols and vitamins [49]. Polyphenols show strong antioxidant activities as ROS scavengers [50]. Polyphenols as an important bioactive compound have been reported to have anticancer effects by upregulating the level of miR-1 [51]. Other reports also show that polyphenol exhibits its biological functions by affecting miRNA-mediated regulation [52]. Thus, FJACP may also affect the level of serum miR-424.

Vitamin C is rich in FJACP which may affect serum levels of microRNAs. Vitamin C is regarded as a reprogramming enhancer for inducing a blastocyst state in embryo cells. Vitamin C demethylates gene promoters by affecting epigenetic modifiers, which activates pluripotency genes and miRNAs of embryo cells [53]. Then, differentiation and development genes are repressed by ESC-enriched miRNAs, which maintain the stem cell state. MicroRNA-expressing profiles have been reported to be affected by vitamin C [54]. Present findings showed that FJACP increased the levels of microRNA-424, which downregulated the activities of Nrf2 and Keap1. The results improved the indices of antioxidant and anti-inflammatory situation of T2DM patients.

According to Chinese theory, FJACP can transfer the strength between deficiency and excess from different parts of the human body, including the upper and lower limbs, the internal organs, and environment. Full body and cooperation among the different organs are the main ideas of FJACP. The limitation of FJACP is that it must be understood well and performed exactly as its central ideas. Overconsumption of FJACP will cause harmful effects on human health since it contains much vitamin C. More trouble, incorrect of FJACP consumption may result in stomach pain.

There were some limitations to the present study. The function of FJACP should be performed in a larger population since it shows fewer side effects. The detail molecular mechanism for the association between proinflammatory cytokines and Keap1and Nrf2 remains unclear. Although FJACP is rich with polyphenols and vitamin C, the relationship between miR-424 and polyphenols or vitamin C remains unknown. To make sure the functional role of FJACP on T2DM is present, much work is still needed to be done in the future.

## 5. Conclusions

Beneficial effects of FJACP were proven here, and the long-term FJACP would be beneficial for improving the symptoms of T2DM. The rehabilitant functions of FJACP might be associated with the increase in the activities and levels of SOD and GSH and the decrease in the levels of ALT and AST, which further increased the level of HDL-c and decreased the level of FJACPG, TC, and LDL-c. All the results decreased oxidative stress in T2DM. Furthermore, FJACP activated Keap1 and Nrf2 via the upregulation of miR-424, which plays an important role in antioxidant and anti-inflammatory activities in T2DM patients. FJACP as a nonpharmaceutical intervention should be developed as a potential way for preventing the risk or development of T2DM.

## Figures and Tables

**Table 1:** Baseline demographic and metabolic characteristics of T2DM subjects.

Characteristics of patients	FJACPG (*n* = 61)	CG (*n* = 61)	t/*χ*^2^	*P* value
Age (years)	56.1 ± 14.4	57.5 ± 12.3	0.24	0.65^a^
Males/females	37/24	39/22	0.14	0.71^b^
Smoker/nonsmoker	18/43	20/41	0.15	0.70^b^
Drinker/nondrinker	24/37	23/38	0.04	0.85^b^
Race, *n* (%)				
Han Zhu	50	48	0.26	0.61^b^
Manchu	8	10	0.26	0.61^b^
Mongolians	2	2	0.26	0.61^b^
Tibetans	1	1	0.51	0.48^b^
Weight (kg)	81.6 ± 17.5	82.8 ± 17.1	0.24	0.51^a^
BMI (kg/m^2^)	29.3 ± 3.6	30.1 ± 2.9	0.64	0.65^a^
HbA1c (%)	8.9 ± 1.6	8.7 ± 1.8	0.71	0.48^a^
FBG (mmol/l)	8.4 ± 1.2	8.7 ± 1.5	0.85	0.32^a^
2hPG (mmol/l)	14.9 ± 2.6	14.1 ± 3.7	0.64	0.56^a^
FJACPG (mmol/l)	2.9 ± 1.2	2.9 ± 1.4	0.13	0.87^a^
TC (mmol/l)	5.8 ± 1.4	5.9 ± 1.6	0.27	0.40^a^
HDL (mmol/l)	1.3 ± 0.3	1.5 ± 0.5	0.24	0.56^a^
LDL (mmol/l)	3.8 ± 1.0	4.0 ± 1.2	0.28	0.71^a^
Resistin (ng/ml)	15.3 ± 4.6	15.5 ± 3.7	1.29	0.14^a^
HOMA-IR	6.4 ± 3.4	6.6 ± 3.5	1.98	0.27^a^
HOMA-IS	66.2 ± 36.7	68.4 ± 27.4	1.58	0.20^a^

Note: ^a^*t*-test and ^b^chi-square test. There was no significantly statistical difference at *P* > 0.05.

**Table 2:** Parameters changes in both groups.

Parameters	FJACPG (*n* = 55)			CG (*n* = 52)			*P* values after 9 months (FJACPG via CG)
	Before	After 9 months	*P* values	Before	After 9 months	*P* values	
Weight (kg)	81.6 ± 17.5	80.5 ± 15.4	0.25	82.8 ± 17.1	80.6 ± 15.3	0.42	0.54
BMI (kg/m^2^)	29.3 ± 3.6	27.1 ± 3.9	0.16	30.1 ± 2.9	28.1 ± 2.6	0.34	0.46
FBG (mmol/l)	8.4 ± 1.2	8.0 ± 1.3	0.21	8.7 ± 1.5	8.3 ± 1.2	0.28	0.39
2hPG (mmol/l)	14.9 ± 2.6	14.0 ± 3.2	0.18	14.1 ± 3.7	13.9 ± 3.4	0.29	0.32
HbAlc (%)	8.9 ± 1.6	8.4 ± 1.9	0.08	8.7 ± 1.8	8.5 ± 1.6	0.43	0.38
FJACPG (mmol/l)	2.9 ± 1.2	2.4 ± 1.4	0.04	2.9 ± 1.4	2.7 ± 1.8	0.31	0.07
TC (mmol/l)	5.8 ± 1.4	5.0 ± 1.1	0.02	5.9 ± 1.6	5.5 ± 2.2	0.04	0.02
HDL (mmol/l)	1.3 ± 0.3	1.6 ± 0.5	0.01	1.5 ± 0.5	1.7 ± 0.2	0. 24	0.17
LDL (mmol/l)	3.8 ± 1.0	3.3 ± 1.2	0.03	4.0 ± 1.2	3.2 ± 1.6	0.01	0.14
Resistin (ng/ml)	15.3 ± 4.6	14.1 ± 3.7	0.27	15.5 ± 3.7	14.5 ± 4.1	0.18	0.35
HOMA-IR	6.4 ± 3.4	6.2 ± 3.9	0.35	6.6 ± 3.5	6.4 ± 2.9	0.14	0.44
HOMA-IS	66.2 ± 36.7	69.4 ± 21.3	0.11	68.4 ± 27.4	70.34 ± 14.2	0.29	0.54

Note: there is no significantly statistical difference at *P* > 0.05.

**Table 3:** Biochemical parameters of enzyme activities.

		SOD (U/ml)	GSH (ng/ml)	ALT (U/ml)	AST (U/ml)
Before	CG	25.34 ± 3.17	26.89 ± 4.19	46.88 ± 12.24	112.34 ± 25.26
	FJACPG	22.78 ± 3.02	25.65 ± 4.04	48.31 ± 11.38	114.18 ± 22.39
	*P* value	0.23	0.56	0.14	0.40
3-month	CG	26.38 ± 2.90	27.45 ± 4.27	45.84 ± 15.23	106.35 ± 28.43
	FJACPG	25.31 ± 3.24	29.36 ± 4.78	43.22 ± 16.51	101.48 ± 27.55
	*P* value	0.64	0.12	0.08	0.17
6-month	CG	25.44 ± 2.37	26.99 ± 4.91	43.24 ± 16.98	102.48 ± 29.36
	FJACPG	30.32 ± 3.24	35.38 ± 4.56	38.46 ± 17.25	96.23 ± 26.22
	*P* value	0.02^∗^	0.04^∗^	0.04^∗^	0.11
9-month	CG	29.49 ± 2.93	32.11 ± 5.27	40.35 ± 18.29	98.40 ± 27.44
	FJACPG	46.39 ± 3.89	39.71 ± 4.56	30.28 ± 19.16	91.95 ± 28.32
	*P* value	0.01^∗^	0.02^∗^	0.01^∗^	0.07

Note: FJACPG: the group treated with the fruit juice of *Actinidia chinensis* Planch.; CG: control group. ^∗^*p* < 0.05 versus CG. Note: *t*-test and chi-square test. There is no significantly statistical difference at *P* > 0.05.

**Table 4:** Comparison of lipid pattern in T2DM patients before and after therapy.

		TG (mmol/l)	TC (mmol/l)	HDL-C (mmol/l)	LDL-C (mmol/l)	MDA (mmol/l)
Before	FJACPG	2.8 ± 1.2	5.7 ± 1.2	1.3 ± 0.3	3.8 ± 1.0	1.7 ± 0.3
	CG	2.7 ± 1.1	5.6 ± 1.2	1.2 ± 0.2	4.0 ± 1.1	1.6 ± 0.2
	*P* value	0.73	0.86	0.64	0.76	0.89
3-month	FJACPG	2.6 ± 1.3	5.5 ± 1.1	1.4 ± 0.2	3.5 ± 1.1	1.5 ± 0.2
	CG	2.7 ± 1.3	5.7 ± 1.1	1.3 ± 0.2	3.9 ± 1.1	1.7 ± 0.3
	*P* value	0.34	0.22	0.18	0.26	0.27
6-month	FJACPG	2.2 ± 1.3	5.1 ± 1.1	1.6 ± 0.2	3.2 ± 1.1	1.3 ± 0.2
	CG	2.8 ± 1.3	5.8 ± 1.1	1.3 ± 0.2	4.0 ± 1.2	1.8 ± 0.3
	*P* value	0.02^∗^	0.04^∗^	0.04^∗^	0.03^∗^	0.02^∗^
9-month	FJACPG	1.8 ± 1.1	4.6 ± 1.4	1.6 ± 0.3	3.0 ± 1.1	0.9 ± 0.1
	CG	2.6 ± 1.3	5.5 ± 1.1	1.2 ± 0.2	3.9 ± 1.2	1.6 ± 0.3
	*P* value	0.01^∗^	0.02^∗^	0.01^∗^	0.01^∗^	0.01^∗^

Note: ^∗^*P* < 0.05 via CG.
